# 3D Printing Technique-Improved Phase-Sensitive OTDR for Breakdown Discharge Detection of Gas-Insulated Switchgear

**DOI:** 10.3390/s20041045

**Published:** 2020-02-14

**Authors:** Zhen Chen, Liang Zhang, Huanhuan Liu, Peng Peng, Zhichao Liu, Shi Shen, Na Chen, Shenhui Zheng, Jian Li, Fufei Pang

**Affiliations:** 1 Key Laboratory of Specialty Fiber Optics and Optical Access Networks, Joint International Research Laboratory of Specialty Fiber Optics and Advanced Communication, Shanghai Institute for Advanced Communication and Data Science, Shanghai University, Shanghai 200444, China; cz_zhenchen@163.com (Z.C.); liangzhang@shu.edu.cn (L.Z.); hhliu@shu.edu.cn (H.L.); noahnss@163.com (Z.L.); shenshi_shu@163.com (S.S.); na.chen@shu.edu.cn (N.C.); 2 State Grid Shanghai Municipal Electric Power Company, 310 South of Chongqing Road, Shanghai 200025, China; pengwyw@163.com (P.P.); zshshdl@163.com (S.Z.); lijianlucy@163.com (J.L.)

**Keywords:** optical fiber sensor, φ-OTDR, 3D-printing technology, breakdown discharge detection, acoustic emission

## Abstract

In this paper, we propose and demonstrate a gas-insulated switchgear (GIS) breakdown discharge detection system based on improved phase-sensitive optical time domain reflectometry (φ-OTDR) assisted by 3D-printed sensing elements. The sensing element is manufactured by a material with a high Poisson ratio for enhancement of the sensitivity of φ-OTDR to the acoustic emission detection during the breakdown discharge process. In our experiment, seven 3D-printed sensing elements incorporating with optical fibers are attached tightly onto the shell of the GIS, which are monitored by φ-OTDR to localize and detect the acoustic emission signal resulted from the breakdown discharge. Ultimately, thanks to the phase demodulation, acoustic signals induced by the breakdown discharge process can be captured and recovered. Furthermore, the time delay analysis of detected signals acquired by different sensing elements on the GIS breakdown discharge unit is able to distinguish the location of the insulation failure part in the GIS unit. It suggests that the φ-OTDR incorporated with 3D printing technology shows the advantage of robustness in GIS breakdown discharge monitoring and detection.

## 1. Introduction

High-voltage (HV) cable and gas insulated switchgear (GIS) are the part and parcel of HV power supply and transmission system whilst their health structure monitoring is always vital for the HV power system [1]. The unavoidable insulation defects in the HV equipment generated during manufacturing, transportation, installation, or operation can lead to insulation degradation [2,3], resulting in partial discharge (PD) which then accelerates and intensifies the insulation failure. The insulation failure eventually leads to the breakdown discharge of the HV equipment, which will cause severe damage to the insulation and crash down the power supply and transmission system. The alternating current (AC) voltage withstand test to the HV equipment in advance is an effective means to detect the hidden insulation defects of the equipment. Therefore, the breakdown discharge detection and localization are necessary to minimize the equipment damages not only in the process of AC voltage withstand test, but also during the formal operation of the HV equipment in power supplies and transmission systems.

Different physical properties, accompanying with the occurrence of the breakdown discharge, have been used to monitor and diagnose the GIS [4], including high-frequency electrical resonance [5], light emission [6], acoustic emission [7], ozone formation, and the release of nitrous oxide gases [8]. In order to detect the electrical resonance, optical signals or gas releases, ultra-high frequency (UHF) sensors, fluorescence fiber or gas sensors must be installed inside the HV devices [9,10]. The installation would make destructions to the surfaces of the devices. Different from above intrusive methods, acoustic emission detection is a nonintrusive detection technology. When the breakdown discharge occurs, there is an instantaneous release of electrical energy, which partially converts to mechanical energy and emits acoustic waves. Then, the generated acoustic wave transmits along with the shell and spreads to the surrounding space of the device, providing a detectable signal for breakdown discharge monitoring. The conventional piezoelectric sensor has already been widely used in acoustic emission detection of the breakdown discharge because of its high sensitivity, good stability, and mature technique [11]. However, as an electrical device, the piezoelectric sensor can be seriously affected by electromagnetic interference, particularly for HV devices, such as HV cables, transformers, and the GIS. The complex electromagnetic environment would inevitably cause a false alarm of an electrical sensing system. Compared to the piezoelectric sensor, the fiber optic sensor has no conductive parts and, therefore, it can be deployed under high electric potential or intense electric fields. For example, optical fibers were wound on the outer surface of the HV equipment shell to measure the acoustic emission signals induced by the PD process with a moderate sensitivity [12]. To enhance the sensitivity, Rohwetter *et al*. proposed a fiber optic sensor with a cylindrical core made of silicone rubber to detect the acoustic emission signals [13]. It has been demonstrated that interferometer-based acoustic emission sensing system incorporated with a cylindrical elastomer wound by optical fibers shows higher sensitivity to acoustic emission signal than the conventional piezoelectric transducer (PZT) [14,15,16]. Note that, most of the previous studies on acoustic emission detection systems are based on single-point fiber optic sensors, which would be inflexible in terms of multiplexing capability for breakdown discharge monitoring multiple objects as well as breakdown discharge events’ locating.

Regarding the breakdown discharge detection requirement of large-scale HV equipment, fiber Bragg grating (FBG)-based discharge detection system are proposed [17,18]. However, the multiplexing of multiple FBGs would increase complexity of the detection system. Compared to the FBG-based detection technique, phase-sensitive optical time-domain reflectometry (φ-OTDR) turns out to be an attractive approach for breakdown discharge-induced acoustic wave detection and exhibits superiority in monitoring large-scale complex networks of GIS systems. The φ-OTDR system, utilizing off-the-shelf optical fiber, does not require sophisticated fabrication setups. In 2018, Cheng Shi proposed a vibration detection system based on the φ-OTDR and the optical fiber Michelson interference to detect the acoustic emission in gas insulated line (GIL) [19]. Ten-ring optical fibers about 15 m were wound on the outer surface of the GIS shell to measure vibration signal of shell induced by flashover. In 2019, Qian Che demonstrated an improved φ-OTDR system with a weak fiber Bragg grating array for acoustic emission detection in cross-linked polyethylene 10-kV power cables [20]. The acoustic emission detection based on φ-OTDR system with highly sensitive cylindrical elastomers shows great advantages in the large-scale PD and breakdown discharge detection of HV equipment. In 2016, Rohwetter combined the silicone rubber sensors with C-OTDR to detect acoustic emission in a 40-KV cable and use phase-resolved diagrams to analyzes the signal [21]. Since the transmission loss of the proposed sensing element in the φ-OTDR system is basically identical to that of optical fiber, which is much smaller than that of FBGs. Thus, problems caused by FBG multiplexing, such as low detected light power, are not of great concern, and the enhanced sensing element can be arbitrarily multiplexed along sensing fiber in φ-OTDR system for a flexible demodulation.

In this paper, we experimentally demonstrate a GIS breakdown discharge detection based on φ-OTDR incorporated with 3D-printed cylindrical elastomer sensing elements. The sensing element is manufactured in a hollow structure with a material of high Poisson rate to increase the sensitivity to acoustic emission signal. A tip-tip electrode in the GIS discharge defect simulation device is used to generate breakdown discharge phenomenon. Multiple sensing elements are manufactured and attached to the shells of three GIS units. The sensing elements on the outer surface of the GIS breakdown discharge unit shell are able to localize the breakdown discharge accurately by intensity localization. Ultimately, phase differential demodulation is implemented to retrieve the acoustic emission signal caused by the breakdown discharge in the GIS. As the pulse repetition rate of the φ-OTDR is 10 kHz, the time delay of detected signals acquired by three sensing elements on the outer surface of the GIS breakdown unit shell is able to further localize the part of insulation failure inside the GIS devices. This suggests that the proposed GIS breakdown discharge detection system with enhanced sensitivity shows great advantage in anti-electromagnetic interference and the large-scale detection range, paving way for practical application in HV power systems.

## 2. 3D-Printing Sensing Element Design

When the breakdown discharge occurs, some part of the released electric energy converts to mechanical energy and emits acoustic waves which transmit to the shell of the GIS. While the optical fiber sensor is embedded in the shell of GIS, the perturbation induced by the acoustic waves will impose onto the optical fiber sensor mostly through the photo-elastic effect. Therefore, the acoustic emission signal could be retrieved by demodulating the intensity or the phase change of the optical fiber sensor.

The optical fiber sensor is proposed to detect the acoustic emission signal generated by breakdown discharge in the GIS. The conventional method is to directly wind the single mode fiber around the outer surface of the GIS shell tightly. In our work, we propose a cylindrical elastomer wound by bend-insensitive optical fiber to serve as the sensing element for the sensitivity enhancement in breakdown discharge detection.

Since the sensing element is attached on the outer surface of the GIS shell, the bottom of the cylindrical elastomer is subjected to acoustic pressure waves caused by the vibration on the surface, and its lateral deformation imposes the strain on the optical fiber and then causes a change in the length of the optical fiber. For the cylindrical elastomer, the relationship between the axial deformation and the lateral deformation can be written as [22]:

(1) $$\varepsilon^{\prime }=𝜇\varepsilon$$

where $\varepsilon^{\prime }$ is lateral deformation which can be written as $\varepsilon^{\prime }=\frac{\Delta D}{D}$ (where *D* is the lateral diameter of the cylindrical elastomer and ΔD is the variation of the lateral diameter), 𝜇 is the Poisson ratio of the material and ε is axial deformation which can be written as:

(2) $$\varepsilon =\frac{F}{EA}$$

where *F* is the axial pressure, *E* is the Young modulus of the material, and *A* is the cros-sectional area (CSA) of the sensing element. Therefore, the variation of lateral diameter ΔD can be written as:

(3) $$\Delta D=\frac{𝜇FD}{EA}$$

Since the optical fiber is wound around the cylindrical elastomer, the variation of the lateral deformation will lead to the change in optical fiber length of the sensing element. The large change of the optical fiber length is the key to improve the sensitivity of the sensing element. Therefore, in order to achieve a larger variation in the lateral deformation, we propose a hollow structure to reduce the CSA of the cylindrical elastomer. The material of cylindrical elastomer is selected to be the flexible filament with a high Poisson ratio. The small CSA of the cylindrical elastomer and the high Poisson’s ratio of the material are able to improve the sensitivity of the sensing element, according to Equation (3).

In addition, the fiber bending loss should be considered in designing the diameter of the cylindrical elastomer. A cylindrical elastomer with a small diameter could increase the transmission loss of the optical fiber wound around it and affect the result of interference light detection. The diameter of the groove in the middle of the cylindrical elastomer is designed as 25 mm.

**Figure 1:**
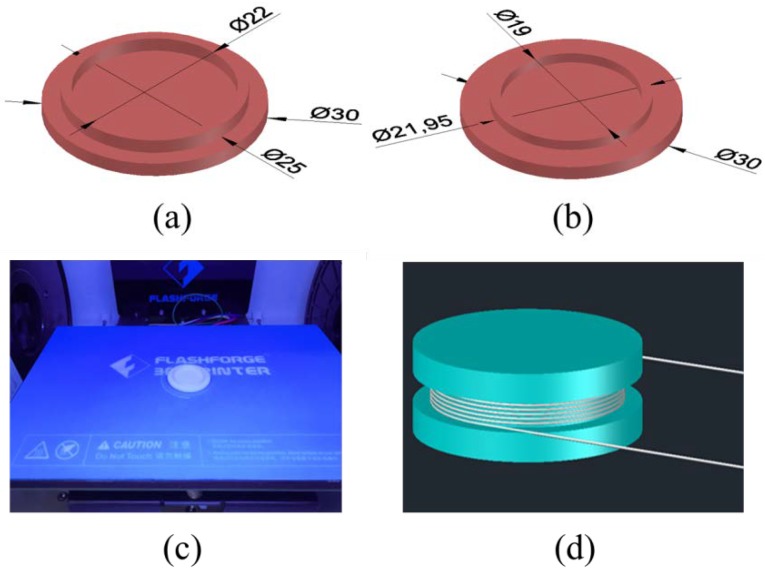
Diagram of (**a**) the external and (**b**) internal component of the cylindrical elastomer; (**c**) the 3D printing process of the cylindrical elastomer; and (**d**) the sensing element spooling with optical fibers.

The cylindrical elastomer is composed of internal and external components, as shown in Figure 1a,b). The bases of both components are designed as cylinders with diameter of 30 mm and height of 3 mm. The joint of external component is designed as a cylindrical ring with outer diameter of 25 mm, inner diameter of 22 mm and height of 3 mm. The joint of internal component is designed as a cylindrical ring with outer diameter of 21.95 mm, inner diameter of 19 mm and height of 2.5 mm. Here, we use a commercial 3D printing machine (FlashForge Dreamer, Jinhua, China) to manufacture the cylindrical elastomer, as shown in Figure 1c. The temperature of the extruder and the platform is set to be 220 °C and 50 °C, respectively. The height of each 3D printing layer is 0.14 mm. The 3D printing process of one single cylindrical elastomer lasts for 55 min.

After being manufactured, the two components of the cylindrical elastomer are connected through the joint. Bend-insensitive optical fiber is chosen to be wound into the groove for 25 turns, as shown in Figure 1d. Then the silicon rubber glue (NANDA 704 RTV Silicone, Changzhou, China) is coated on the wound fiber to fix the fiber firmly. The transmission loss of one single sensing element is approximately 0.1 dB.

## 3. Experimental Setup

**Figure 2:**
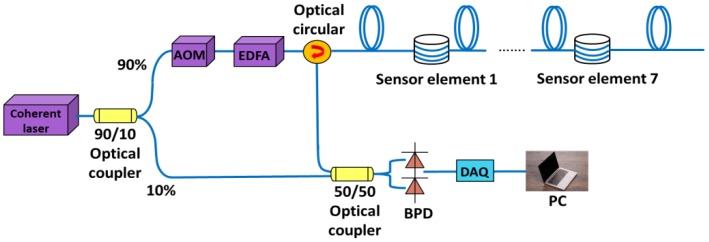
Experimental setup of a φ-OTDR sensing system incorporating with 3D-printed sensing elements: acoustic-optic modulator (AOM); erbium-doped fiber amplifier (EDFA); balanced photo- detector (BPD); data acquisition card (DAQ); and personal computer (PC).

As shown in Figure 2, the φ-OTDR system is used to demodulate the acoustic signals detected by sensing elements. Coherent lightwave from a narrow linewidth continuous-wave (CW) laser source at 1550 nm is separated into two parts by a 90:10 optical coupler. The 90% part is launched into an acousto-optic modulator (AOM, Gooch and Housego T-M200-0.1C2J-3-F2S, Dowlish Ford, Ilminster, UK) driven by an FPGA. The input light is modulated to pulse light and introduced 200-MHz optical frequency shift by the AOM. An erbium-doped fiber amplifier (EDFA) is used to amplify the light pulse. Seven sensing elements are connected into the sensing fiber in series. As acoustic waves disturb the sensing elements, applied external strains would cause changes of the refractive index of optical fibers as well as the optical path length which, hence, induce phase change of the Rayleigh backscattered lightwaves. After mixed with the 10% part of the input light, the Rayleigh backscattered light is then transferred into the electrical signals by a balanced photo-detector (BPD). A high-speed data acquisition (DAQ) card (AlazarTech ATS9360, 8-bit, 1 GS/s, Pointe-Claire, Canada) triggered by the FPGA acquires the electric signal. Signal processing including I/Q demodulation and phase differential calculation is implemented by a personal computer (PC). In our experiments, we manufacture seven sensing elements. Each of the two adjacent sensors is connected by a 50 m single mode fiber. The total length of the sensing fiber is around 600 m. Accordingly, the pulse width and pulse repetition rate are set to be 25 ns and 10 kHz, respectively. The spatial resolution of the φ-OTDR system is determined by the pulse width, which is 2.5 m in our case, while the spatial resolution of the GIS breakdown discharge detection system mainly depends on the deployed positions of the sensing elements since each of the 3D-printed sensing element is served as a quasi-point sensor.

**Figure 3:**
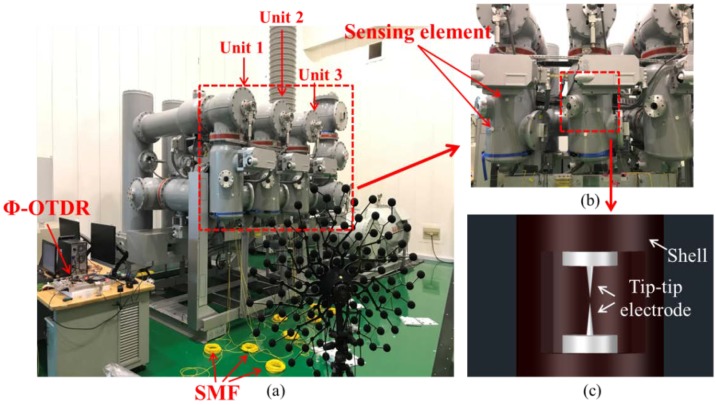
(**a**) GIS discharge defect simulation device; (**b**) attachments of the sensing elements on the outer surface of GIS shell; (**c**) and tip–tip electrode inside the GIS discharge defect simulation device.

The GIS discharge defect simulation device (Shanghai Electric Power Research Institute, Shanghai, China) is used to simulate the PD and breakdown discharge phenomenon in the process of GIS AC voltage withstand test, as shown in Figure 3a. The chambers of GIS are filled with SF6 as insulation and arc extinguishing medium. The metal tip defects model inside the GIS consists of a tip-tip electrode, a single-phase circuit breaker, a single-phase isolated switch, a tip-tip electrode distance adjustment device and a built-in UHF sensor. The schematic diagram of the tip-tip electrode is shown in Figure 3c. The tip-tip electrode inside the GIS shell is able to simulate the PD and breakdown discharge caused by metal tip defects in the GIS. The distance between two tip electrodes and the AC voltage imposed on the tip-tip electrode can be adjusted by the control board. The maximum voltage imposed on the tip-tip electrode could reach up to 252 kV. All the sensing elements are attached to the outer surfaces of the GIS shells tightly with tapes, as shown in Figure 3b.

**Figure 4:**
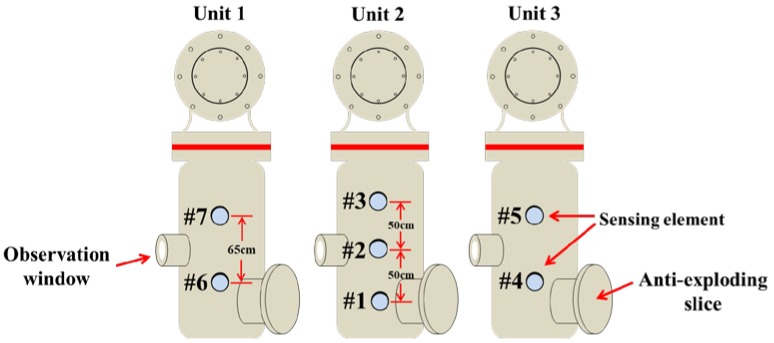
Deployment of sensing elements on the shell of GIS discharge defect simulation device.

There are three isolated GIS units in the simulation device. Each GIS unit is able to work independently. In the experiments, the metal tip defects module in GIS unit 2 is used to generate the breakdown discharge phenomenon. Three sensing elements are attached on the outer surface of GIS unit 2 shell tightly with tapes. Sensing element #2 is attached in the middle of outer surface of GIS shell, which is the closest to the tip–tip electrode. Sensing element #1 is attached 50 cm below sensing element #2, and sensing element #3 is attached 50 cm above sensing element #2, as shown in Figure 4. Sensing elements #4 and #5 are attached to the outer surface of GIS unit 3 shell, while sensing elements #6 and #7 is attached to the outer surface of GIS unit 1 shell. After the deployment, the voltage imposed on the tip-tip electrode starts to rise gradually.

## 4. Experimental Results and Discussions

**Figure 5:**
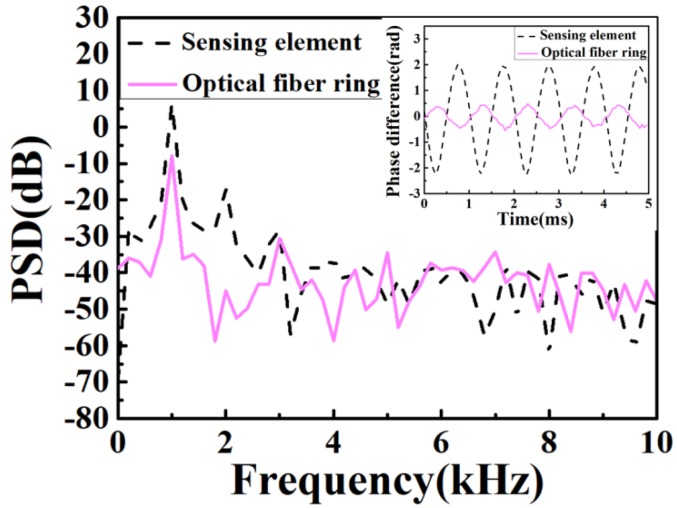
PSD comparison of acoustic detection with the sensing element and the optical fiber ring at 10 Vpp 1000 Hz vibration. (The inset shows the recovered detected acoustic signals).

Firstly, the sensing response of the φ-OTDR system with the 3D-printed sensing element for acoustic wave detection has been tested, compared with that of conventional optical fiber. We embedded a sensing element on a piezo buzzer which is excited by a signal generator. The signal generator generates the sine wave with the frequency of 1000 Hz and the peak-to-peak voltage amplitude of 10 V. As a comparison, we also directly wound the bend insensitive optical fiber into a ring without the cylindrical elastomer. The diameter and number of turns are the same as that of the sensing element. The optical fiber ring is embedded on the piezo buzzer with the same vibration frequency and peak-to-peak amplitude. As shown in the inset of Figure 5, the peak-to-peak phase difference value of the detected signal of the 3D printed sensing element is 4 rad. The detected signal of the optical fiber ring shows a peak-to-peak phase difference value of 0.8 rad, which is much less than that of sensing element with cylindrical elastomer under the same experimental conditions. Corresponding power spectral density (PSD) spectra are shown in Figure 5. The peak at the frequency of 1000 Hz of the sensing element with cylindrical elastomer is approximately 15 dB higher than that of the optical fiber ring. The experimental results show that the cylindrical elastomer-based 3D printed sensing element is able to significantly improve the sensitivity of the acoustic wave detection as well as the signal-to-noise ratio by one order of magnitude.

Then, the φ-OTDR system with 3D-printed sensing element is utilized to perform the GIS breakdown discharge detection. In the experiments, the breakdown discharge occurs when the AC high voltage imposed on the tip-tip electrode reaches up to about 45 kV. It is worth noting that the proposed fiber optic sensing system for the breakdown discharge detection is able to work without any interference caused by heavy electromagnetic pulse during the occurrence of the breakdown discharge, which shows the anti-electromagnetic interference of the system.

**Figure 6:**
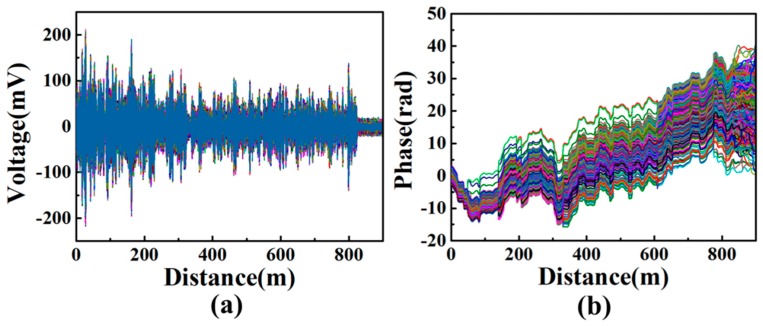
(**a**) Original superposed signals acquired by the DAQ; (**b**) demodulated phase traces after the I/Q demodulation.

The I/Q demodulation is implemented to demodulate signals acquired by DAQ card [23,24]. As shown in Figure 6a, original signals acquired by DAQ include 200 MHz frequency shift generated by the AOM. By the orthogonal demodulation method and the phase unwrap technique, the in-phase (*I*) and the quadrature (*Q*) components of the beat signals are obtained for the calculation of the amplitude ($\left|E\left(t\right)\right|\;\propto \sqrt{I^{2}\;+\;Q^{2}}$) and the phase ($\varphi \left(t\right)=tan^{-1}\left(\frac{I}{Q}\right)+2k\pi \;$), respectively [24]. Correspondingly, the demodulated phase distributions diffuse randomly from trace to trace, as shown in Figure 6b. It mainly results from either the short-term frequency drift of the laser source or the frequency instability of the AOM driving signal. Thanks to the phase differential method [24], the accurate phase change induced by external vibration can be obtained by calculating the phase difference between two neighboring locations.

**Figure 7:**
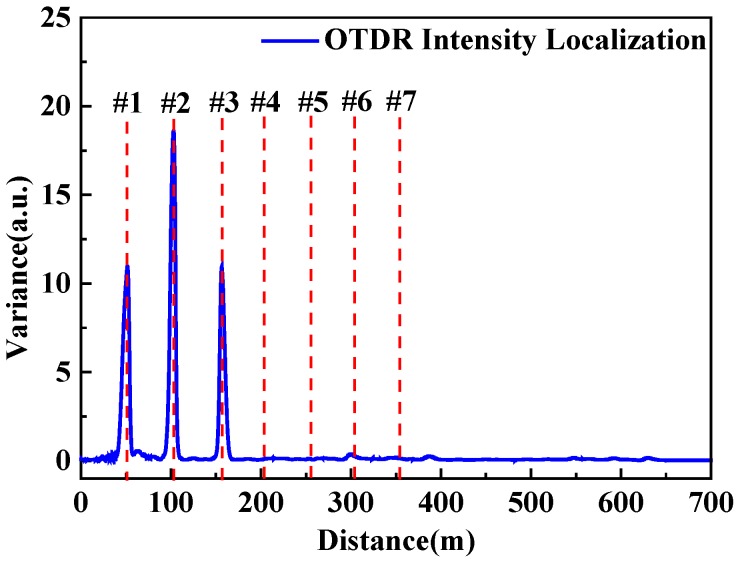
Intensity localization signal of breakdown discharge.

Through the calculation of the standard deviation of the obtained amplitude at each fiber location, intensity localization can be realized, as shown in Figure 7. There are three obvious localizing peaks at the location of the three sensing elements on the outer surface of GIS unit 2 shell, which include sensing elements #1, #2 and #3. Meanwhile, there is no localizing peak at the location of the other sensing elements, which means the breakdown discharge in a GIS unit would not affect the localization signals of sensing elements attached on the outer surface of other GIS unit shells. The intensity localization method of the acoustic emission detection system is able to localize the GIS unit which breakdown discharge occurs inside accurately.

**Figure 8:**
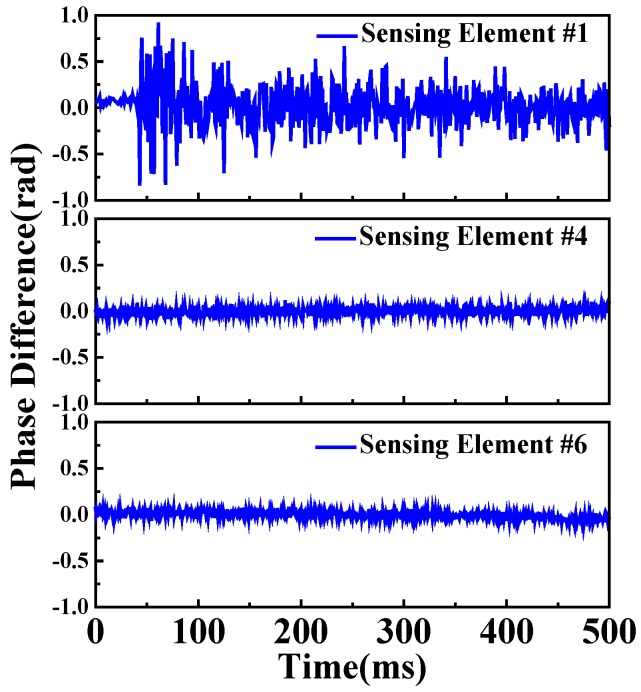
Detected signals of sensing elements #1, #4, and #6 at a pulse repetition rate of 2 kHz.

The phase differential calculation is implemented to eliminate the influences caused by phase noise and retrieve the acoustic wave signal acquired by the sensing elements [24,25]. Figure 8 shows the phase differential calculation, in which the differential distance is set to be 30 m. The detected signals of sensing element #1 on the outer surface of GIS unit 2 shell, sensing element #4 on the outer surface of GIS unit 3 shell and sensing element #6 on the outer surface of GIS unit 1 shell at the pulse repetition rate of 2 kHz are shown in Figure 8. The acoustic emission signal acquired by sensing element #1 lasts for 500 ms and keeps decaying. The sensing element on the outer surface of GIS unit 2 shell is able to detect the breakdown discharge signal, while only background noise signals are acquired by the sensing elements on the outer surface of the other GIS units shells. Therefore, the localization signal and detected signal of the sensing element would not be affected by the acoustic emission wave generated by breakdown discharge in the GIS units, which could minimize the possibility of false alarms and localization errors in breakdown discharge monitoring.

**Figure 9:**
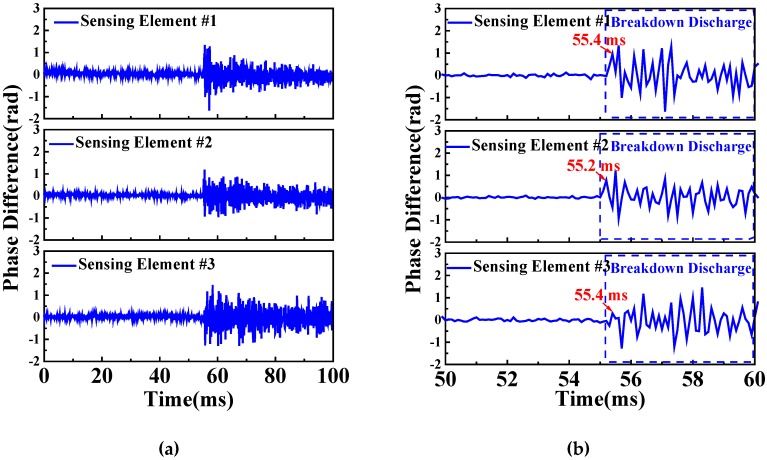
Detected signals of sensing elements #1, #2, and #3 at a pulse repetition rate of 10 kHz from (**a**) 0–100 ms and (**b**) 50–60 ms

In order to analyze the higher frequency of vibration signal generated by breakdown discharge, the pulse repetition rate is set to be 10 kHz and the GIS AC voltage withstand test is repeated. The acquisition time of a complete detected signal is 100 ms. The detected signals of sensing elements #1, #2 and #3 attached on the outer surface of GIS unit 2 shell are shown in Figure 9a. The breakdown discharge occurs at a time of 55 ms, approximately. Therefore, the detected signals of sensing elements #1, #2, and #3 from 50 to 60 ms are shown in Figure 9b. The first peak of the detected acoustic emission signal of sensing element #1 is at 55.4 ms, which is the same as the one of sensing element #3. The first peak of the detected acoustic emission signal of sensing element #2 is at 55.2 ms, which is 0.2 ms earlier than that of sensing elements #1 and #3. This means that the acoustic emission wave transmits to the sensing element which is attached closest to the breakdown discharge region firstly. Through analyzing the time delay of start points of the acoustic emission signals detected by multiple sensing elements attached on the outer surface of the same GIS unit shell, the accurate location of the breakdown discharge and damaged part of the GIS can be found. The sensing element that acquires acoustic emission signal earliest is the one closet to breakdown discharge. Considering the distance between sensing elements #1 and #2 is around 50 cm and the time delay between the signals acquired by sensing elements #1 and #2 is measured as 0.2 ms, the propagation speed of the vibration wave transmitted on the GIS shell is able to be calculated as 2500 m/s, which is close to the propagation speed of the sound wave in metal.

**Figure 10:**
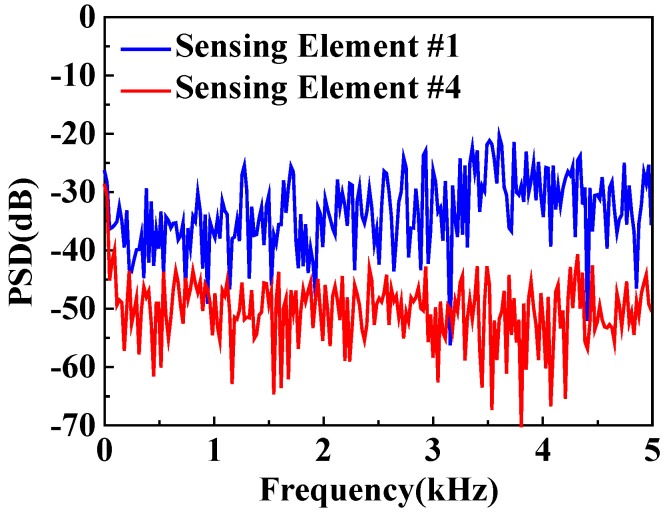
Power spectral density (PSD) comparison of detected signals acquired by the sensing elements #1 and #4 at a pulse repetition rate of 10 kHz.

**Figure 11:**
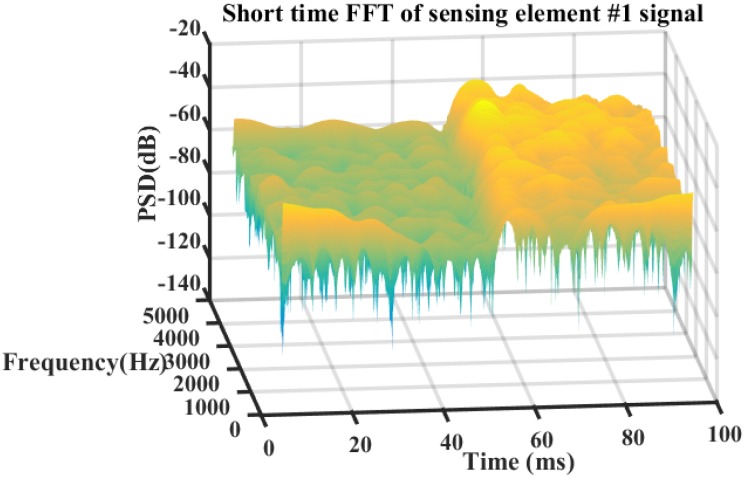
Short time FFT spectra of the detected signal acquired by sensing element #1 at a pulse repetition rate of 10 kHz.

The frequency information of the detected signals is analyzed by a Fast Fourier Transform (FFT) calculation. The pulse repetition rate of the φ-OTDR is 10 kHz. The frequency spectrum of detected signals acquired by sensing elements #1 and #4 are shown in Figure 10. The power of the detected signals of sensing element #1 is larger than that of sensing element #4 at the frequency from 0 to 5 kHz, which means the acoustic emission signal generated by breakdown discharge is a broadband signal. The short time FFT calculation is implemented to the detected signals of sensing element #1, as shown in Figure 11. The size of the hamming window is set to be 100 sample points. The signal before 55 ms is background noise, which is low in power. The power of the acoustic emission signal after 55 ms is higher. Additionally, the power of the acoustic emission signal keeps decaying at the full detecting frequency band, which means the breakdown discharge is an instantaneous event.

## 5. Conclusion

In this paper, we propose a GIS breakdown discharge detection based on φ-OTDR incorporated with 3D printed cylindrical elastomer sensing elements. The hollow structure and high Poisson rate material of the sensing element are able to increase lateral deformation under longitudinal pressure, which leads to high sensitivity to vibration. Seven sensing elements are manufactured and connected into φ-OTDR in series. Breakdown discharge detection in the process of GIS AC voltage withstand testign has been achieved on a GIS discharge defect simulation device with pulse repetition rates of 2 kHz and 10 kHz, respectively. Results show that the positions of all the sensing elements on the outer surface of the GIS breakdown discharge unit shell appear obvious localizing peaks, indicating that the breakdown discharge to the GIS unit can be successfully located. By phase demodulation, the detected signals of the sensing elements are able to retrieve the acoustic emission signal generated during the breakdown discharge. Furthermore, through the time delay of the detected signals acquired by different sensing elements at different locations on the GIS systems, the accurate localization of the insulation failure part of the GIS unit can be realized. The advantages, such as the efficient deployment, large-scale detection and the anti-electromagnetic interference, etc., enable the system to be more suitable for breakdown discharge detection in the process of GIS AC voltage withstand testing and formal operation. Moreover, the cylindrical elastomer-based element not only benefits the sensitivity improvement of optical fiber in φ-OTDR system but also for other fiber sensors, e.g., the FBG. This suggests that the proposed 3D-printed sensing element can be also incorporated with the common FBG to further improve its sensitivity. Consequently, it is believed that the 3D-printing technique definitely offers a flexible approach to optimize conventional fiber sensors, particularly for customized requirement in a number of practical scenarios.
